# Relationship between the autonomic nervous system and cerebral autoregulation during controlled breathing

**DOI:** 10.1007/s00421-025-05933-9

**Published:** 2025-08-18

**Authors:** Agnieszka Uryga, Monika Najdek, Piotr Urbański, Magdalena Kasprowicz, Teodor Buchner

**Affiliations:** 1 Department of Biomedical Engineering, Wroclaw University of Science and Technology, Wybrzeze Wyspianskiego 27, 50-370 Wroclaw, Poland; 2 Clinical Department of Anaesthesiology and Intensive Therapy, Faculty of Medicine, Wroclaw Medical University, Wroclaw, Poland; 3 Faculty of Physics, Warsaw University of Technology, Warsaw, Poland

**Keywords:** Autonomic nervous system, Cerebral autoregulation, Arterial blood pressure, Cerebral blood velocity, Joint symbolic dynamics

## Abstract

**Introduction:**

Controlled breathing is a hemodynamic maneuver known to influence both baroreflex and chemoreflex sensitivity. This study investigated the impact of respiratory-driven oscillations on the relationship between cerebral autoregulation and autonomic nervous system (ANS) activity.

**Methods:**

Sixty-one volunteers (median age: 23 years) underwent noninvasive measurements of arterial blood pressure (ABP), cerebral blood velocity (CBv), end-tidal CO_2_ (EtCO_2_), and respiratory rate during spontaneous breathing and during three 5-min sessions of controlled breathing at 6, 10, and 15 bpm. Cerebral autoregulation was assessed using transfer function analysis by calculating phase shift (PS) and gain between ABP and CBv in the very low frequency (VLF; 0.02–0.07 Hz) and breathing frequency (BF; [0.1, 0.17, 0.25] ± 0.02 Hz) ranges. ANS activity was assessed using baroreflex sensitivity (xBRS), heart rate variability (HRV) metrics in time and frequency domains, and entropy-based parameters. Cardiovascular coupling was assessed using the joint symbolic dynamics of beat-to-beat pulse interval and systolic blood pressure.

**Results:**

Increasing respiratory rate led to decreased EtCO_2_ (*p* < 0.001), diminished cardiovascular coupling (*p* < 0.01), and reduced systemic ABP control, as indicated by lower normalized low-frequency HRV and xBRS (both *p* < 0.001). A linear mixed-effects model, adjusted for EtCO_2_ and respiratory rate, showed that PS at VLF and BF was modulated by ANS metrics, whereas gain was mainly affected by respiratory parameters, with a nonsignificant contribution from ANS.

**Conclusions:**

Higher respiratory rates reduced cardiovascular coupling, diminished ANS activity, and modified its interaction with cerebral autoregulation. Respiratory parameters should be considered when assessing ANS–cerebral autoregulation relationship.

**Supplementary Information:**

The online version contains supplementary material available at 10.1007/s00421-025-05933-9.

## Introduction

Cerebral autoregulation is a homeostatic mechanism that maintains adequate cerebral blood flow (CBF) in response to fluctuations in mean arterial pressure. While early models of cerebral autoregulation suggested a relatively wide mean arterial pressure range (60–150 mm Hg), within which CBF remains constant (Lassen 1964), more recent studies have challenged this view, indicating that the actual range of CBF constancy is narrower than originally proposed (Brassard et al. 2021; Numan et al. 2014). Since continuous direct monitoring of CBF poses a challenge, cerebral blood velocity (CBv), measured in the middle cerebral artery through transcranial Doppler ultrasound, is commonly used as a surrogate for assessing dynamic cerebral autoregulation (Giller et al. 1993; Newell & Aaslid 1992; Robba et al. 2018). Cerebral autoregulation has been conceptualized as a high-pass filter that primarily operates within the low-frequency range (Burma et al. 2024). While cerebral arterioles cannot respond quickly enough to counteract high-frequency oscillations in arterial blood pressure (ABP), they effectively buffer slower oscillations, being the most effective for < 0.05 Hz (Claassen et al. 2016a; Diehl et al. 1998). Since cerebral autoregulation reflects the dynamic relationship between ABP and CBF (or CBv), transfer function analysis (TFA) has become a widely used method for quantifying this relationship (Claassen et al. 2016a). TFA is characterized by three primary metrics: gain (or magnitude), phase shift (PS), and coherence. Gain reflects the damping effect between ABP and CBv, with lower values indicating more effective autoregulatory control. PS quantifies the time lag between ABP and CBv at a given frequency, with higher PS suggesting better buffering by cerebral vasculature (Claassen et al. 2016b; Kostoglou et al. 2024a, b).

The cerebral pressure–flow relationship is driven by sympathetic, cholinergic, and myogenic mechanisms (Hamner and Tan 2014). Adequate CBF and perfusion depend on the integrated functioning of two regulatory systems: cerebral autoregulation and the baroreflex. These are mediated by cerebrovascular and cardiovascular control mechanisms, respectively (Ogoh et al. 2010; Tzeng et al. 2010). The baroreflex, an autonomic nervous system (ANS) response to changes in blood pressure, operates through a negative feedback loop (Benarroch 2008; Lanfranchi and Somers 2002). It modulates heart rate and systemic vascular resistance to preserve perfusion pressure and ensure continuous blood flow to all vital organs, including the brain (Rickards et al. 2011). The functional dependency between cerebral autoregulation and the ANS function has been investigated in both animal and human studies (Ainslie and Brassard 2014; Goadsby, 2013; Mankoo et al. 2023; Ogoh et al. 2010; Tzeng et al. 2010; Witter et al. 2017; Zhang et al. 2002).

ANS activity is predominantly assessed using heart rate variability (HRV). The utility of HRV analysis in both time and frequency domains has been shown in previous studies (Chand et al. 2024; Pham et al. 2021). However, growing evidence suggests that nonlinear metrics may better capture the complexity and irregularity of cardiovascular dynamics, given the inherently nonlinear coordination of autonomic control (Castiglioni et al. 2023). Joint symbolic dynamics (JSD), a coarse-grained, nonlinear method for time series analysis, has proven effective for assessing baroreflex sensitivity (BRS) and cardiovascular coupling by evaluating the relationship between R–R intervals (from ECG) and systolic blood pressure (SAP) from ABP (Baumert et al. 2005, 2013, 2015; Javorka et al., 2011). In addition, entropy-based measures have been recognized as robust indicators of cardiovascular variability and complexity (Castiglioni et al. 2023).

Controlled breathing is a hemodynamic maneuver known to influence both cerebral autoregulation (Diehl et al. 1995) (Reinhard et al. 2003) (Eames et al. 2004) and ANS activity (Frederiks et al. 2000) (Paprika et al. 2014). Carotid and aortic baroreceptors, along with chemoreceptors, modulate sympathetic nerve activity in response to arterial CO_2_ levels (Huang et al. 1990; Prabhakar 2016). CO_2_ is a key determinant in regulating the ventilation–perfusion ratio, contributing to both efficiency (chemoreflex sensitivity) and stability (periodic breathing) (Francis et al. 2000). During controlled breathing, an external respiratory rhythm is imposed, which may impact arterial CO_2_ levels by driving ventilation beyond the individual’s respiratory set-point. This can modulate baroreceptor and chemoreceptor activity without altering the underlying set-point itself. The breathing pattern and CBv are closely linked by the partial pressure of CO_2_ (PaCO_2_) and chemoreflex activity. Respiratory-frequency oscillations in ABP are directly transmitted to the cerebral vasculature (Sayin et al. 2022). Furthermore, since CBF is highly sensitive to CO_2_ changes, reduced ventilation during slow breathing may elevate CO_2_ levels, leading to vasodilation of cerebral vessels. This, in turn, increases CBF, enhancing CO_2_ clearance from brain tissue, and reduces chemoreceptor drive (Ainslie and Duffin 2009).

In this study, we applied a controlled breathing maneuver in healthy volunteers to modulate cerebral autoregulation through changes in baroreflex and chemoreflex activity. We analyzed the relationship between cerebral autoregulation and ANS metrics using Spearman correlation and a linear mixed-effects model adjusted for respiratory parameters. In addition, ANS activity was assessed using nonlinear metrics that describe the cardiovascular coupling dynamics.

## Materials and methods

### Participants

Two datasets were used in this study. The first dataset (database 1) includes 56 healthy volunteers (32 females, 24 males, median age: 23 years, range: 18–31 years) whose data were collected at the Neuroengineering Laboratory (www.brainlab.pwr.edu.pl), Wroclaw University of Science and Technology, between October 2014 and June 2015, and analyzed retrospectively. The second dataset (database 2) included 37 healthy volunteers (21 females, 16 males, median age: 22 years, range: 18–31 years) whose data were collected prospectively at the same institution between October 2023 and January 2024. There were no methodological differences between the two datasets. Both databases were combined to increase the overall number of participants, enhance statistical power, and improve the generalizability of the results. These datasets were previously used in studies on nonlinear ANS metrics (Uryga et al. 2024) and time–frequency cerebral autoregulation metrics (Uryga et al. 2017). All participants were adults (> 18 years old), non-smokers, with a normal body mass index, free from any chronic illnesses, and not taking any medications on a long-term basis. No medical issues were identified during the medical interview, which was conducted by a physician. Exclusion criteria were: inconsistent breathing with the metronome (defined as a respiratory rate > 1 bpm above or below the set rhythm (*n* = 4), failure to complete the full study protocol (*n* = 1), poor signals quality due to artifacts in either ABP or CBv signals (*n* = 18), and absence of ECG recordings (*n* = 9). A flow chart is presented in Supplementary Fig. S1.

### Ethical approval and informed consent

The study complied with the Declaration of Helsinki of the World Medical Association. Ethical approval for both databases was obtained from the Commission of Bioethics at Wroclaw Medical University, Wroclaw, Poland (database 1: KB–170/2014 and database 2: KB–179/2023/N), before commencing the study. All volunteers provided written informed consent before participation.

### Study protocol

**Fig. 1:**
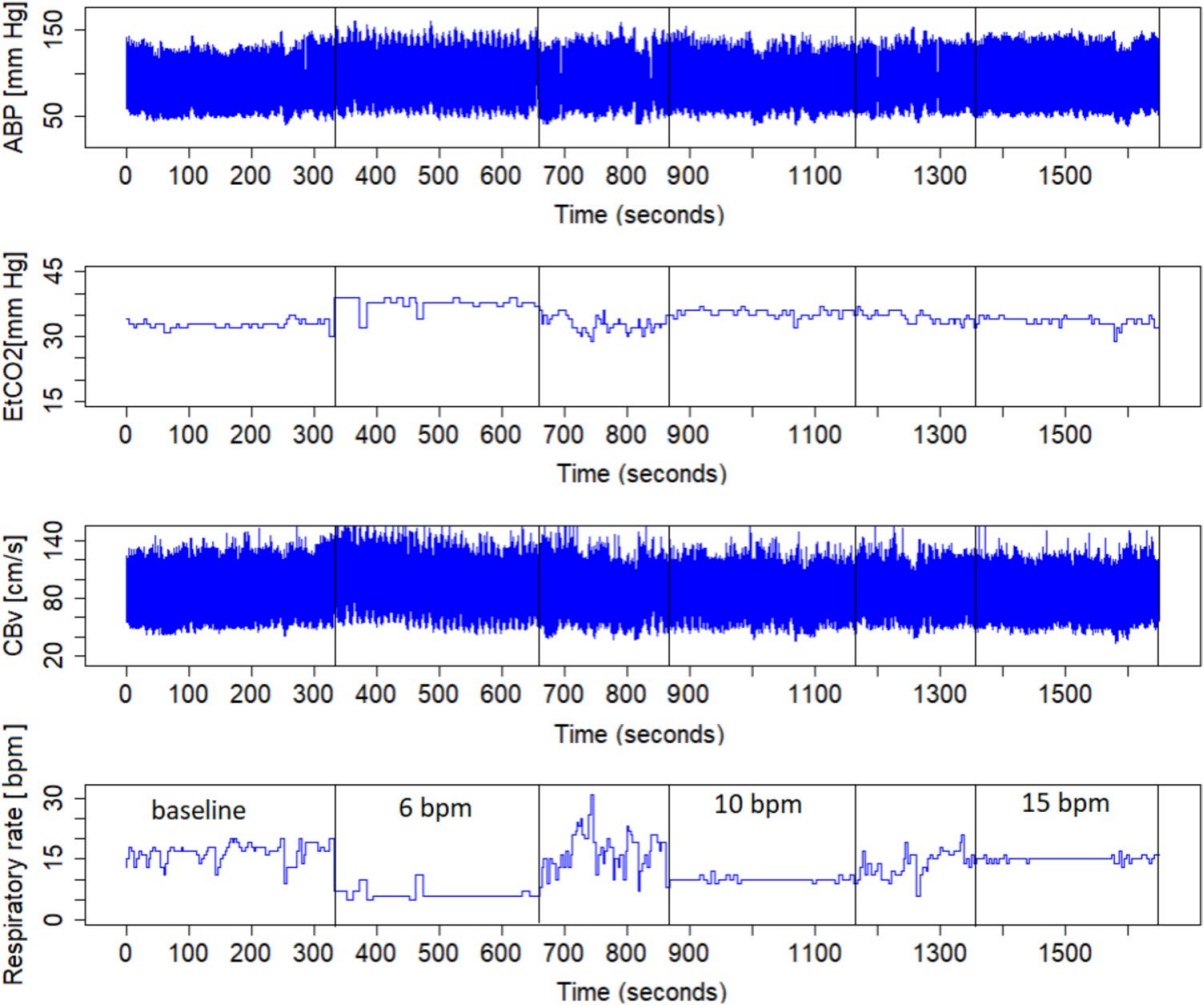
Exemplary time trends of arterial blood pressure (ABP), end-tidal carbon dioxide (EtCO_2_), cerebral blood velocity (CBv), and respiratory rate during spontaneous and controlled breathing maneuver in a volunteer

Volunteers were instructed to refrain from alcohol and caffeine consumption for at least 12 h before the examination. Before the recordings began, participants were given time to stabilize physiologically and psychologically to minimize potential stress responses. Following a 5-min resting period during which participants breathed spontaneously, a controlled breathing session was initiated. This session consisted of three 5-min recordings at respiratory rates of 6, 10, or 15 bpm, corresponding to 0.1 Hz, 0.17 Hz, and 0.25 Hz, respectively. Participants were instructed to avoid deep breathing during the procedure. A mobile metronome application was used to guide breathing rhythm. The application featured a light ball moving back and forth across the screen, with the ball’s position indicating the breathing phase: inspiration when it reached the right edge, and expiration when it reached the left edge. To ensure participants could follow the protocol accurately, each underwent an individual training session before. Respiratory rate was monitored in both databases using capnography, allowing for precise tracking of respiratory rate, CO_2_, and EtCO_2_. Each recording epoch was separated by a short break to allow physiological parameters to return to baseline. All recordings were conducted at room temperature, with external stimuli minimized and under physician supervision. Participants were included in the analysis only if they maintained the targeted respiratory rates of 6, 10, and 15 bpm, with and allowed tolerance of ± 1 bpm. A representative example of the recorded signals is presented in Fig. 1.

### Data acquisition and signal preprocessing

CBv was measured unilaterally in the middle cerebral artery (MCA) using transcranial Doppler ultrasonography (Doppler BoxX, DWL, Compumedics Germany GmbH, Singen, Germany, in database 1; EMS-9PB, Delica, Shenzhen, China, in database 2). A 2 MHz probe attached to a head frame was positioned over the temporal window and fixed at a constant angle to ensure optimal signal acquisition. The MCA was identified on the insonated side at depths of 50 to 55 mm, using reference values of 50 mm for depth and 55 ± 12 cm/s for CBv (Willie et al. 2011). ABP was measured using the Finometer MIDI (FMS Medical Systems, Amsterdam, The Netherlands) in database 1 and the Finapres Nova (FMS Medical Systems, Amsterdam, The Netherlands) in database 2. The cuff was placed on the middle finger of the left hand, which rested on an armrest adjusted to maintain heart level positioning. A three-lead surface electrocardiogram (ECG) was used to record the electrical activity of the heart. Expired end-tidal CO_2_ (EtCO_2_) and respiratory rate were measured via nasal cannula using a portable capnography monitor (RespSense™, NONIN, Plymouth, USA). Volunteers were instructed to breathe through their noses. All signals were recorded at a sampling frequency of 200 Hz using the ICM + system (Cambridge Enterprise Ltd, Cambridge, UK). Preprocessing of respective signals to remove artifacts was performed before any analysis was conducted. Artifacts in CBv and ABP signals were identified and excluded through visual inspection and preprocessing algorithms built-in the Neurokit2 library (Makowski et al. 2021), which includes ectopic peak detection. In addition, the first derivative of the signal was calculated, and if peaks exceeding twice the standard deviation of the derivative were detected within the given timeframe, linear interpolation was applied to correct both ABP and CBv signals.

### Cerebral autoregulation

Transfer function (TFA) analysis was performed using the open-source code provided by Prof. D. Simpson (available at http://www.car-net.org/) in accordance with current guidelines (Claassen et al. 2016b; Panerai et al. 2023). The procedural steps are outlined below. The beat-to-beat interval was derived from ABP signal, used as an alternative to ECG for marking cardiac rhythm (Constant et al. 1999). Systolic peaks were identified as the local maxima of ABP waveform, defined by their temporal position and amplitude. Raw ABP and CBv signals were smoothed and downsampled by averaging over short intervals determined by the systolic peaks. Due to variability in heart rate, the resulting time series were non-uniformly sampled. To address this, spline interpolation was applied at a sampling frequency of 4 Hz, ensuring uniform sampling for subsequent analysis. No detrending was performed on the resampled data segments; however, mean values were subtracted from both signals. TFA of the ABP–CBv relationship was performed using Welch’s method with a Hanning window. Data were segmented into 102.4-s windows with 50% superposition. Negative phase values at frequencies below 0.1 Hz were removed to avoid phase wrapping. A coherence threshold was applied using 95% confidence limits based on the number of windows, and frequencies with low-magnitude-squared coherence were excluded from averaging. TFA metrics were calculated in the 0.02–0.07 Hz frequency range (very low, VLF) and at three breathing frequency (BF) bands, centered at 0.1 Hz, 0.17 Hz, and 0.25 Hz (± 0.02 Hz), corresponding to respiratory rates of 6, 10, and 15 bpm (± 1 bpm), respectively. For spontaneous breathing, the BF range was determined individually for each participant as mean respiratory rate ± standard deviation. The VLF range was chosen to represent the frequency band where cerebral autoregulation is most active. The BF was selected to assess the transmission of forced breathing-induced blood pressure oscillations to the cerebral vasculature, consistent with previous studies (Diehl et al. 1995; Reinhard et al. 2003). The results of the TFA analysis were reported using average coherence, phase shift (PS), and gain. The coherence function allows for the identification of conditions where estimates of gain and phase are reliable. Lower PS and higher gain values are interpreted as indicating worsened cerebral autoregulation. Further interpretation of these metrics is presented in previous studies (Claassen et al. 2021). Examples of frequency-dependent TFA curves for spontaneous and controlled breathing are presented in Supplementary Fig. S2.

### Heart rate variability

HRV metrics were calculated from the ECG signal, following the standards set by the Task Force of the European Society of Cardiology and the North American Society of Pacing and Electrophysiology (Malik et al. 1996). R-peak detection based on ECG was performed according to the method described by Brammer (2020), combined with filtering proposed by Elgendi (2010), which uses a second-order Butterworth filter with an 8–20 Hz frequency band. A detailed description of the formulas used to estimate HRV metrics is presented in the NeuroKit2 library documentation (Makowski et al. 2021; Pham et al. 2021). In the time domain, the following metrics were determined: the standard deviation of the R–R intervals (SDNN), the square root of the mean of the squared successive differences between adjacent R–R intervals (RMSSD), mean R–R intervals (meanNN), and the proportion of R–R intervals differing by more than 20 ms or 50 ms (pNN20 and pNN50, respectively). The power spectral density of HRV was determined by Fast Fourier Transform (FFT) using the Welch periodogram method and determined at low-frequency (LF; 0.04–0.15 Hz) and high-frequency (HF; 0.15–0.4 Hz) ranges. To improve comparability and minimize the impact of the power spectral density estimation method, spectral components were normalized by total power, and reported as normalized low-frequency (LFn) and high-frequency (HFn) components (Bachler 2017). The ratio between low- and high-frequency components (LF/HF) was also computed. Nonlinear, entropy-based HRV measures included four metrics: multiscale entropy (MSEn) (Costa et al. 2002), approximate entropy (ApEn) (Shi et al. 2017), sample entropy (SampEn) (Shi et al. 2017), and fuzzy entropy (FuzzyEn) (Chen et al. 2009). These entropy metrics have been associated with cardiac sympathetic and parasympathetic activity (Castiglioni et al. 2023; Nardelli et al. 2023).

### Heart rate and baroreflex sensitivity

Heart rate (HR) was determined using the FFT as the frequency corresponding to the first harmonic of ABP signal, within the range of 40–140 beats/min (0.67–2.33 [Hz]). Baroreflex sensitivity was estimated from ABP signal based on the cross-correlation baroreflex sensitivity (xBRS) method proposed by Westerhof (Wesseling et al. 2017; Westerhof et al. 2004). xBRS was calculated using the regression between SAP and beat-to-beat pulse intervals over a 10-s sliding window. Only windows where the correlation p-value was less than 0.05 and no ectopic beats were detected were included (Wesseling et al. 2017). Due to the variability in SAP and interbeat intervals, which may cause time shifts between series, the algorithm accounts for optimal delay. In young, healthy adults, this delay typically ranges from 0 to 1 s, though it can extend to 2–3 s depending on the balance between vagal and sympathetic activity (Wesseling et al. 2017). In our study, the dominant time delay was consistently 2 s across the cohort and did not vary significantly with respiratory rate.

### Assessment of cardiovascular coupling

Cardiovascular coupling was estimated from ABP signal using the JSD technique. Symbolic representations of beat-to-beat changes in pulse intervals (R–R intervals) and SAP are aligned. Thus, a change in the SAP affects the subsequent R–R interval. This allows for effective embedding of baroreflex-related R–R dynamics. A detailed description of the algorithm is presented in the Supplementary Materials. Here, we present only a summary. A bivariate vector *X* of length *n*, containing time series data of R–R intervals and SAP, is transformed into a bivariate symbol sequence *S* as follows:

1 $$S=\{{\left[{s}_{n}^{R-R},{s}_{n}^{SAP} \right]}^{T}\}$$

where s $\in \{\mathrm{0,1}\}$. Hence, ${s}_{n}^{R-R}$ is defined as 1 when ${x}_{n}^{R-R}-{x}_{n+1}^{R-R}<{l}^{R-R}$, and ${s}_{n}^{SAP}$ is defined as 1 when ${x}_{n}^{SAP}-{x}_{n+1}^{SAP}<{l}^{SAP}$. Otherwise, ${s}_{n}^{R-R}$ and ${s}_{n}^{SAP}$ are defined as 0. In the following, the threshold values are set to zero (${l}^{R-R}$ = 0, ${l}^{SAP}$= 0). Using a word length of three, this method produces 64 possible word types, which are compiled into matrix *W*. This provides a statistically sufficient representation of cardiovascular dynamics over up to 30 min of beat-to-beat signals (Baumert et al. 2015). A schematic representation of the transformation from vector *X* to matrix *W* is shown in Supplementary Fig. 3. The *W* matrix enables the derivation of the relative frequency of baroreflex-like word types (JSD_sym_) and patterns opposing baroreflex behavior (JSD_diam_) (Baumert et al. 2015). JSD_sym_ and JSD_diam_ have been established as sensitivity measures for quantifying cardiovascular responsiveness to different stress maneuvers (Baumert et al. 2013; Kabir et al. 2013).

### Statistical analysis

The normality of the data distribution was assessed using the Kolmogorov–Smirnov test with Lilliefors correction. As the normality assumption was rejected for most variables, nonparametric tests were used. To assess differences in physiological parameters, cerebral autoregulation indices, and ANS metrics during controlled breathing, the Friedman ANOVA test for repeated measurements was used. Effect sizes were estimated using Kendall’s *W*. Post-hoc comparisons (*m* = 3) were performed using the Wilcoxon signed-rank test for the following pairwise comparisons: 6 versus 10 bpm; 6 versus 15 bpm; and 10 versus 15 bpm. The Bonferroni correction was applied to adjust for increased Type I error, setting the post-hoc significance level at *α*_post-hoc_ = α/m, with a corrected threshold of *α*_post-hoc_ < 0.017. Comparisons of physiological parameters, cerebral autoregulation indices, and ANS metrics between spontaneous breathing and each respective respiratory rate were also performed using the Wilcoxon signed-rank test. The relationships between ANS metrics and cerebral autoregulation during both spontaneous and controlled breathing were assessed using Spearman correlation. A linear mixed-effects model with participants as random effects and respiratory rate, EtCO_2_, and the respective ANS metrics as fixed explanatory variables, was used to explain variation in cerebral autoregulation parameters across the three combined respiratory rates. This method is recommended for analyzing datasets with repeated observations, especially when the number of observations varies between conditions or when data are not normally distributed (Schielzeth et al. 2020). The significance level was set as 0.05. Data are presented as medians with interquartile range unless stated otherwise. Statistical analyses were performed using STATISTICA 13 (Tibco, Palo Alto, USA) and R Statistical Software (v.4.0.2; R Foundation for Statistical Computing, Vienna, Austria).

## Results

### Physiological parameters

Sixty-one participants were included in the analysis (36 (59%) female; median age: 23 ± 3 years, range: 18–31 years). The physiological parameters are summarized in Table 1. The median respiratory rate during spontaneous breathing was 15 ± 6 bpm. Compared with spontaneous breathing, CBv decreased at all respective respiratory rates (*p* < 0.001 for all), ABP increased at both 10 bpm (*p* < 0.001) and 15 bpm (*p* < 0.001), and HR increased significantly at 15 bpm (*p* = 0.004). EtCO_2_ at 10 bpm and 15 bpm was significantly lower compared to baseline (*p* < 0.001 for both). With increasing respiratory rate, EtCO_2_ progressively decreased (*p* < 0.001), while HR (*p* < 0.001) and ABP (*p* < 0.001) increased. No significant changes were observed in CBv across controlled breathing.

**Table 1:** Physiological parameters in the total group during spontaneous breathing (baseline) and controlled breathing. Data are presented as median values ± interquartile range

	Respiratory rate					
Parameter	baseline	6 bpm	10 bpm	15 bpm	*p*^a^	Effect size^b^
CBv [cm/s]	68.3 ± 20.1^+$~^	55.9 ± 21.7	57.7 ± 20.9	57.1 ± 22.2	0.367	NA
ABP [mm Hg]	86.1 ± 20 ^$~^	85.9 ± 18.5	92.5 ± 19.1^#^	92.8 ± 24.8^#^	** < 0.001**	0.21
HR [beats/min]	73.6 ± 14.5^~^	70.6 ± 14.3	77.2 ± 12.2^#^	78.6 ± 11.3^#^	** < 0.001**	0.16
EtCO_2_ [mm Hg]	36.6 ± 5.8^$~^	36.5 ± 8.0	33.8 ± 7.7^#^	32.6 ± 8.1^#&^	** < 0.001**	0.50

*Abbreviations:* CBv, cerebral blood velocity; ABP, mean arterial blood pressure; EtCO_2_, end-tidal carbon dioxide; HR, heart rate

^a^Nonparametric Friedman’s ANOVA comparing controlled breathing at 6, 10, and 15 bpm; significant values are shown in bold

^b^Effect size was estimated using Kendall’s W; NA refers to not available for nonsignificant results; ANOVA post-hoc tests were performed using Wilcoxon test where: ^#^ versus 6 bpm; ^&^ versus 10 bpm; comparisons between baseline and respective respiratory rates were performed using the Wilcoxon test where: ^+^ versus 6 bpm; ^$^ versus 10 bpm; ^~^ versus 15 bpm

### Changes in cerebral autoregulation metrics during controlled breathing

The average values of coherence, PS, and gain during spontaneous and controlled breathing are presented in Table 2. In the VLF range, coherence was higher at both 6 bpm (*p* < 0.001) and 10 bpm (*p* < 0.001) compared to spontaneous breathing, with no significant differences observed between controlled respiratory rates. PS was significantly higher at 10 bpm (*p* < 0.001) and 15 bpm (*p* < 0.001) compared to spontaneous breathing, and increased with higher respiratory rates (*p* = 0.025). No significant changes in gain were observed in the VLF. In the BF range, coherence was higher at all controlled respiratory rates compared to spontaneous breathing (*p* < 0.001 for all), with no differences observed among the controlled respiratory rates. PS increased at both 6 and 10 bpm (*p* < 0.001) compared to spontaneous breathing, but significantly decreased with increasing respiratory rate (*p* < 0.001). Gain was lower at 6 bpm (< 0.001) compared to spontaneous breathing and increased with higher respiratory rate (*p* < 0.001). A linear mixed-effects model, adjusted for EtCO₂ and respiratory rate, showed that EtCO₂ significantly influenced all TFA metrics in both the VLF and BF ranges (Supplementary Table S1).

**Table 2:** Cerebral autoregulation and autonomic nervous system metrics during spontaneous breathing (baseline) and controlled breathing. Data are presented as median values ± interquartile range

Cerebral autoregulation	Respiratory rate					
VLF	Baseline (*n* = *56*)	6 bpm (*n* = *56*)	10 bpm (*n* = *54*)	15 bpm (*n* = *47*)	*p*^a^	Effect size *w*^*b*^
Coherence [a.u.]	0.45 ± 0.17^+$^	0.54 ± 0.16	0.54 ± 0.21	0.51 ± 0.26	0.496	NA
PS [°]	47.6 ± 25.3^$~^	55.6 ± 26.8	63.7 ± 24.0^#^	72.8 ± 34.1	**0.025**	0.09
Gain [cm s^−1^ mmHg^−1^]	0.52 ± 0.20^~^	0.45 ± 0.20	0.49 ± 0.20	0.43 ± 0.34	0.065	NA

BF	Baseline (*n* = *57*)	6 bpm (*n* = *61*)	10 bpm (*n* = *61*)	15 bpm (*n* = *61*)	*p*^a^	Effect size *w*^*b*^
Coherence [a.u.]	0.88 ± 0.11^+$~^	0.93 ± 0.10	0.95 ± 0.08	0.97 ± 0.04	0.102	NA
PS [°]	12.7 ± 18.1^+$^	40.1 ± 28.6	30.7 ± 19.2^#^	8.3 ± 20.5^#&^	** < 0.001**	0.32
Gain [cm s^−1^ mmHg^−1^]	1.03 ± 0.39^+^	0.81 ± 0.40	1.08 ± 0.40^#^	1.18 ± 0.48^#^	** < 0.001**	0.41

Autonomic nervous system	Respiratory rate					
	baseline (*n* = *61*)	6 bpm (*n* = *61*)	10 bpm (*n* = *61*)	15 bpm (*n* = *61*)	*p*^a^	Effect size *w*^*b*^
SDNN [ms]	63 ± 25^+^	99 ± 35	63 ± 28^#^	51 ± 29^#&^	** < 0.001**	0.81
RMSSD [ms]	47 ± 31^+^	70 ± 31	51 ± 39^#^	37 ± 39^#&^	** < 0.001**	0.45
MeanNN [ms]	800 ± 137	800 ± 146	769 ± 149^#^	764 ± 138^#^	** < 0.001**	0.17
pNN20 [%]	59 ± 22^+^	67 ± 15	56 ± 32^#^	53 ± 37^#&^	** < 0.001**	0.19
pNN50 [%]	20 ± 27^+^	36 ± 25	23 ± 33^#^	12 ± 33^#&^	**0.003**	0.22
xBRS [ms/mm Hg]	9.8 ± 5.3^+$^	14.1 ± 11.3	11.1 ± 6.7^#^	9.2 ± 7.7^#&^	** < 0.001**	0.33
LFn [a.u.]	0.40 ± 0.21^+$~^	0.85 ± 0.09	0.23 ± 0.17^#^	0.23 ± 0.17^#&^	** < 0.001**	0.79
HFn [a.u.]	0.30 ± 0.22^+$~^	0.08 ± 0.06	0.64 ± 0.27^#^	0.40 ± 0.34^#&^	** < 0.001**	0.90
LF/HF [a.u.]	1.32 ± 1.62^+$^	11.13 ± 7.74	0.35 ± 0.56^#^	0.77 ± 1.57^#&^	** < 0.001**	0.89
JSD_sym_[a.u.]	0.32 ± 0.11^$~^	0.32 ± 0.15	0.24 ± 0.13^#^	0.29 ± 0.16^&^	**0.002**	0.09
JSD_diam_[a.u.]	0.02 ± 0.02^+$^	0.03 ± 0.04	0.02 ± 0.04^#^	0.02 ± 0.03^#^	** < 0.001**	0.14
SampEn [a.u.]	1.4 ± 0.5^+$^	0.9 ± 0.3	1.2 ± 0.4^#^	1.5 ± 0.4^#&^	** < 0.001**	0.84
MSEn [a.u.]	1.4 ± 0.3^+^	1.1 ± 0.3	1.4 ± 0.4^#^	1.4 ± 0.2^#&^	** < 0.001**	0.31
ApEn [a.u.]	1.2 ± 0.2^+$~^	0.8 ± 0.2	1.0 ± 0.1^#^	1.1 ± 0.1^#&^	** < 0.001**	0.61
FuzzyEn [a.u.]	1.1 ± 0.3^+^	0.9 ± 0.2	1.1 ± 0.2^#^	1.1 ± 0.4^#&^	** < 0.001**	0.64

*Abbreviations*: PS, phase shift; VLF, very low frequency (0.02–0.07 Hz), BF, breathing frequency (determined individually for spontaneous breathing and fixed at 0.10; 0.17; 0.25 Hz ± 0.02 Hz for controlled breathing); SDNN, standard deviation of the R–R intervals; RMSSD, square root of the mean of the squared successive differences between adjacent R–R intervals; MeanNN, mean intervals between normal R-peaks; pNN20 and pNN50, the percentage of adjacent R–R intervals that differ by more than 20 ms or 50 ms, respectively; LFn, HFn, normalized power spectral density of the R–R interval time series in the low-frequency range (LF, 0.04–0.15 Hz) and the high-frequency range (HF, 0.15–0.40 Hz), obtained by dividing the respective power spectra by a total power (TP, 0.04–0.40 Hz); LF/HF; low-to-high-frequency ratio; MSEn, multiscale entropy; ApEn, approximate entropy; SampEn, sample entropy, FuzzyEn, fuzzy entropy; xBRS, baroreflex sensitivity; JSD_sym_, the relative frequency of baroreflex-like word types; JSD_diam_, the relative frequency of patterns that are opposed to baroreflex behavior

^a^Nonparametric Friedman’s ANOVA comparing controlled breathing at 6, 10, and 15 bpm;  significant values are shown in bold

^b^Effect size was estimated using Kendall’s W; NA refers to not available for nonsignificant results; ANOVA post-hoc tests were performed using Wilcoxon test where: ^#^ versus 6 bpm; ^&^ versus 10 bpm; comparison between baseline and respective respiratory rate was performed using Wilcoxon test where: ^+^ versus 6 bpm; ^$^ versus 10 bpm; ^~^ versus 15 bpm;

### Changes in ANS metrics during controlled breathing

ANS metrics during spontaneous and controlled breathing at 6, 10, and 15 bpm are presented in Table 2. Compared to spontaneous breathing, the most pronounced changes in ANS metrics were observed at 6 bpm. With increasing respiratory rate, significant reductions were observed in all of the time-domain metrics (*p* < 0.01 for all metrics). In the frequency domain, LFn decreased with higher respiratory rate (*p* < 0.001), while HFn was the lowest at 6 bpm and increased with respiratory rate (*p* < 0.001). All entropy metrics (SampEn, MSEn, ApEn, and FuzzyEn) significantly increased (*p* < 0.001). A linear mixed-effects model, adjusted for mean EtCO_2_ and respiratory rate, showed that EtCO_2_ remained a significant factor influencing the ANS metrics (Supplementary Table S2).

### Cardiovascular coupling

**Fig. 2:**
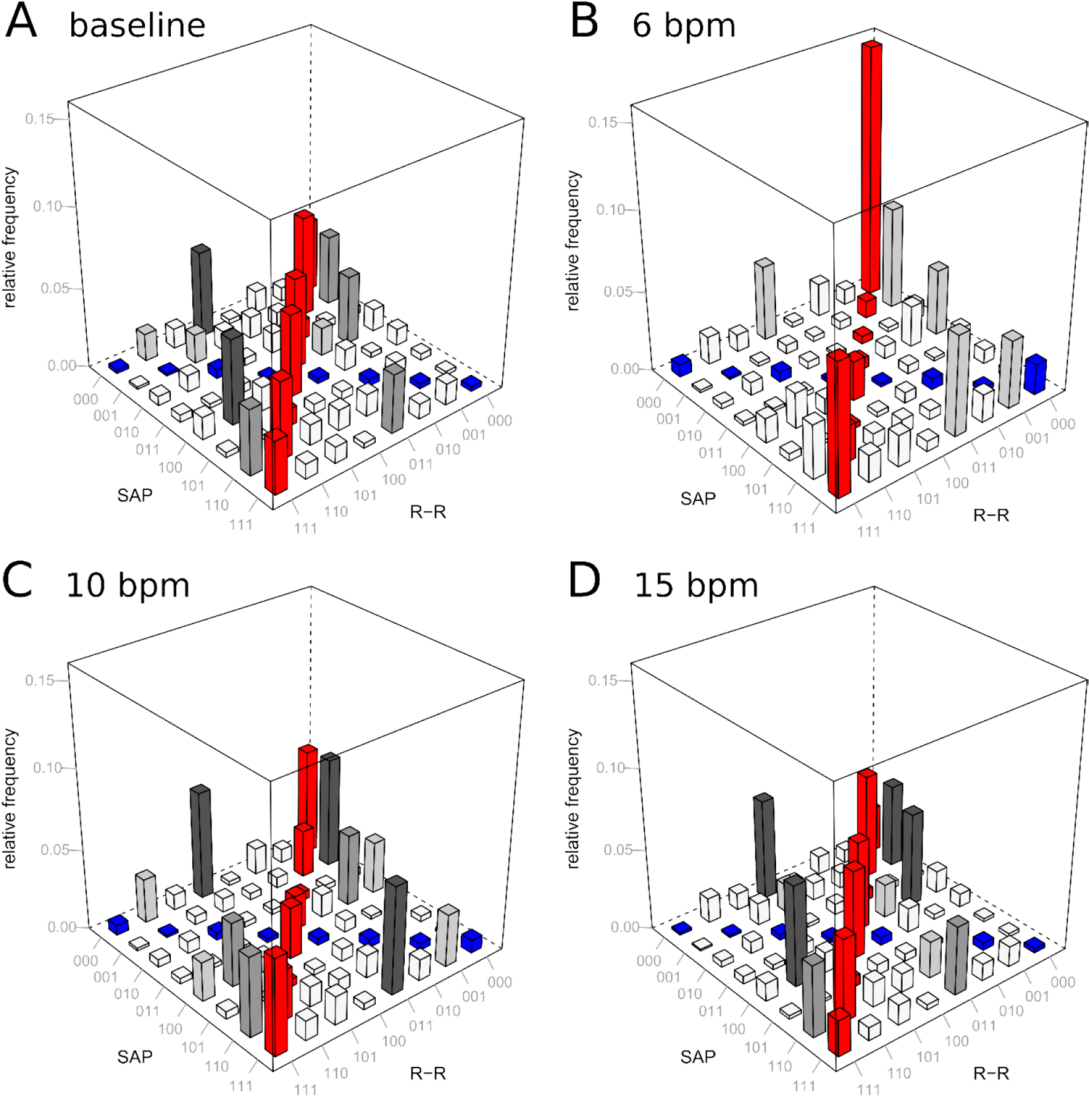
Word distribution matrices (*W*) of cardiovascular joint symbolic dynamics (JSD) are shown for **A** spontaneous breathing (baseline) and controlled breathing at frequencies of **B** 6 bpm, **C** 10 bpm, and **D** 15 bpm, for the total group. Relative frequency is visualized using a color scale; red for JSD_sym_ and blue for JSD_diam_. The indices JSD_sym_ and JSD_diam_ correspond to the main diagonal and antidiagonal of the matrix *W*, respectively. Both the relative frequency of baroreflex-like word types (JSD_sym_) and the relative frequency of patterns that are opposed to baroreflex behavior (JSD_diam_) significantly decreased. These observations may reflect that higher respiratory rates are associated with reduced cardiovascular oscillations. Consequently, the symbolic word matrix indicates that the system becomes more random and exhibits a more stochastic nature. Abbreviations: SAP—systolic blood pressure, R–R—the duration of a ventricular cardiac cycle measured between two successive R waves

Word distribution matrices (*W*) for cardiovascular coupling using JSD are shown in Fig. 2. Higher breathing frequencies were associated with a significant decrease in JSD_sym_ (*p* = 0.002) and JSD_diam_ (*p* < 0.001) word types, suggesting reduced baroreflex activity and a shift toward more stochastic cardiovascular behavior. A linear mixed-effects model, adjusted for mean EtCO_2_ and respiratory rate, demonstrated that EtCO_2_ remained a significant factor influencing the JSD indices (Supplementary Table S2).

### Relationship between ANS activity and cerebral autoregulation

**Fig. 3:**
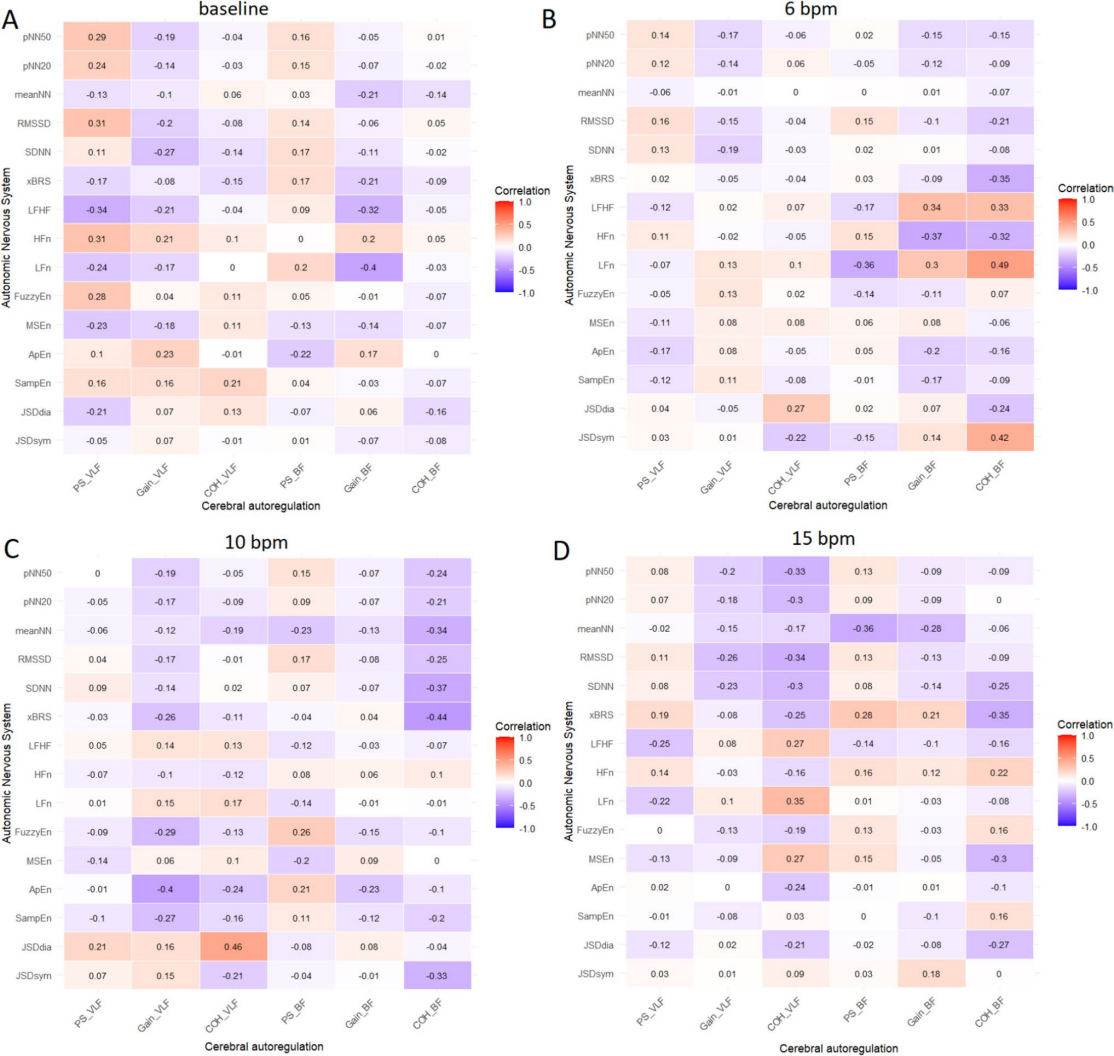
The matrix of Spearman correlations for autonomic nervous system (vertical axis) and cerebral autoregulation metrics (horizontal axis) during **A** spontaneous breathing (baseline) and controlled breathing at frequencies of **B** 6 bpm, **C** 10 bpm, and **D** 15 bpm for the total group. *Abbreviations*: PS, phase shift; COH, coherence; VLF, very low frequency (0.02–0.07 Hz), BF, breathing frequency (determined individually for spontaneous breathing and fixed at 0.10; 0.17; 0.25 Hz ± 0.02 Hz for controlled breathing); SDNN, standard deviation of the R–R intervals; RMSSD, square root of the mean of the squared successive differences between adjacent R–R intervals; pNN20 and pNN50, the percentage of adjacent R–R intervals that differ by more than 20 ms or 50 ms, respectively; LFn, HFn, normalized power spectral density of the R–R interval time series in the low-frequency range (LF, 0.04–0.15 Hz) and the high-frequency range (HF, 0.15–0.40 Hz), obtained by dividing the respective power spectra by a total power (TP, 0.04–0.40 Hz); LF/HF; low-to-high-frequency ratio; MSEn, multiscale entropy; ApEn, approximate entropy; SampEn, sample entropy, FuzzyEn, fuzzy entropy; xBRS, baroreflex sensitivity; JSD_sym_, the relative frequency of baroreflex-like word types; JSD_diam_, the relative frequency of patterns that are opposed to baroreflex behavior

The analysis of the relationship between ANS activity and cerebral autoregulation was performed using two approaches: a direct method based on Spearman correlation (Fig. 3) and a novel approach, using a linear mixed-effects model (Table 3).

**Table 3:** Linear mixed-effects model explaining the variation in cerebral autoregulation metric phase shift (PS) by autonomic nervous system (ANS) metrics, adjusted for respiratory parameters: end-tidal CO_2_ (EtCO_2_) and respiratory rate. Significant factors are indicated in bold.

		ANS		Respiratory rate (bpm)		EtCO_2_ (mm Hg)	
Cerebral autoregulation	Metric	F	p	F	p	F	p
PS VLF [^°^]	SDNN [ms]	12.2	** < 0.001**	43.5	** < 0.001**	1.1	0.294
	RMSSD [ms]	9.2	** < 0.001**	35.1	** < 0.001**	6.7	**0.010**
	MeanNN [ms]	17.5	** < 0.001**	10.2	**0.002**	0.9	0.339
	pNN20 [%]	10.7	** < 0.001**	30.7	** < 0.001**	2.3	**0.128**
	pNN50 [%]	6.2	**0.014**	33.9	** < 0.001**	6.2	**0.014**
	xBRS [ms/mm Hg]	11.9	**0.001**	35.8	** < 0.001**	8.3	**0.005**
	LFn [a.u.]	2.5	0.119	32.2	** < 0.001**	5.1	**0.026**
	HFn [a.u.]	8.9	0.297	17.9	** < 0.001**	30.6	** < 0.001**
	LF/HF [a.u.]	0.8	0.381	28.6	** < 0.001**	13.6	** < 0.001**
	JSD_sym_ [a.u.]	2.9	0.093	25.8	** < 0.001**	12.2	**0.001**
	JSD_dia_ [a.u.]	0.1	0.731	31.4	** < 0.001**	23.1	** < 0.001**
	MSEn [a.u.]	2.3	0.130	7.6	**0.007**	5.9	**0.016**
	ApEn [a.u.]	15.8	** < 0.001**	0.1	0.736	0.2	0.695
	SampEn [a.u.]	1.8	0.181	6.9	**0.010**	1.8	0.181
	FuzzyEn [a.u.]	6.0	**0.016**	6.7	**0.011**	6.0	**0.015**
PS BF [^°^]	SDNN [ms]	20.1	** < 0.001**	1.0	0.323	1.1	0.298
	RMSSD [ms]	18.2	** < 0.001**	> 0.1	0.836	6.9	**0.009**
	MeanNN [ms]	15.5	** < 0.001**	3.2	0.074	0.1	0.727
	pNN20 [%]	13.0	** < 0.001**	> 0.1	0.843	3.7	0.057
	pNN50 [%]	8.5	**0.004**	> 0.1	0.807	15.9	** < 0.001**
	xBRS [ms/mm Hg]	11.0	**0.001**	> 0.1	0.994	13.1	** < 0.001**
	LFn [a.u.]	16.9	** < 0.001**	3.7	0.057	1.2	0.269
	HFn [a.u.]	0.5	0.479	0.6	0.811	33.3	** < 0.001**
	LF/HF [a.u.]	4.2	**0.044**	1.3	0.259	11.7	**0.001**
	JSD_sym_ [a.u.]	6.1	**0.015**	0.1	0.738	12.0	**0.001**
	JSD_dia_ [a.u.]	< 0.1	0.890	< 0.1	0.892	29.0	** < 0.001**
	MSEn [a.u.]	9.3	**0.003**	4.3	**0.039**	4.3	**0.039**
	ApEn [a.u.]	22.0	** < 0.001**	12.0	**0.001**	0.2	0.685
	SampEn [a.u.]	4.0	**0.048**	1.6	0.209	19.5	** < 0.001**
	FuzzyEn [a.u.]	16.3	** < 0.001**	5.6	**0.019**	3.7	0.057

*Abbreviations*: VLF, very low frequency (0.02–0.07 Hz), BF, breathing frequency (determined individually for spontaneous breathing and fixed at 0.10; 0.17; 0.25 Hz ± 0.02 Hz for controlled breathing); SDNN, standard deviation of the R–R intervals; RMSSD, square root of the mean of the squared successive differences between adjacent R–R intervals; pNN20 and pNN50, the percentage of adjacent R–R intervals that differ by more than 20 ms or 50 ms, respectively; LFn, HFn, normalized power spectral density of the R–R interval time series in the low-frequency range (LF, 0.04–0.15 Hz) and the high-frequency range (HF, 0.15–0.40 Hz), obtained by dividing the respective power spectra by a total power (TP, 0.04–0.40 Hz); LF/HF; low-to-high-frequency ratio; MSEn, multiscale entropy; ApEn, approximate entropy; SampEn, sample entropy, FuzzyEn, fuzzy entropy; xBRS, baroreflex sensitivity; JSD_sym_, the relative frequency of baroreflex-like word types; JSD_diam_, the relative frequency of patterns that are opposed to baroreflex behavior

Spearman correlation analyses between ANS activity and cerebral autoregulation during spontaneous and controlled breathing are presented in Fig. 3 A-D. During spontaneous breathing (Fig. 3A), the strongest correlations were observed between PS at VLF and pNN50 (*r*_*S*_ = 0.29), RMSSD (*r*_*S*_ = 0.31), HFn (*r*_*S*_ = 0.31), FuzzyEn (*r*_*S*_ = 0.28), and a reciprocal correlation with LF/HF (*r*_*S*_ = − 0.34). Gain at VLF correlated negatively with SDNN (*r*_*S*_ = − 0.27), while other correlations were negligible. PS at BF showed a negative correlation with ApEn (*r*_*S*_ = − 0.22), with remaining associations negligible. Gain at BF correlated negatively with LF/HF (*r*_*S*_ = − 0.32) and LFn (*r*_*S*_ = − 0.40). At 6 bpm (Fig. 3B), neither PS nor gain at VLF showed significant correlations with ANS metrics. PS at BF correlated negatively with LFn (*r*_*S*_ = − 0.36), while gain correlated positively with LF/HF (*r*_*S*_ = 0.34) and LFn (*r*_*S*_ = 0.30), and negatively with HFn (*r*_*S*_ = − 0.37). At 10 bpm (Fig. 3C), PS at VLF showed no significant correlation with ANS metrics. Gain showed significant negative correlations with xBRS (*r*_*S*_ = − 0.26), FuzzyEn (*r*_*S*_ = − 0.29), ApEn (*r*_*S*_ = − 0.40), and SampEn (*r*_*S*_ = − 0.27). Neither PS nor gain at BF showed significant correlations with ANS metrics. At 15 bpm (Fig. 3D), PS at VLF correlated negatively with LF/HF (*r*_*S*_ = − 0.25), while gain correlated negatively with RMSSD (*r*_*S*_ = − 0.26). PS at BF correlated positively with xBRS (*r*_*S*_ = 0.28) and negatively with meanNN (*r*_*S*_ = − 0.36). Gain at BF showed negative correlation with meanNN (*r*_*S*_ = − 0.28).

Linear mixed-effects model analysis between ANS metrics and cerebral autoregulation during controlled breathing is presented in Table 3. A linear mixed-effects model adjusted for EtCO_2_ and respiratory rate demonstrated that PS at the VLF was significantly modulated by time-domain ANS metrics (SDNN, *p* < 0.001; RMSSD, *p* < 0.001; meanNN, *p* < 0.001; pNN20, *p* < 0.001; pNN50, *p* = 0.014; xBRS, *p* = 0.001) and selected HRV entropy metrics (ApEn, *p* < 0.001; FuzzyEn, *p* = 0.016). Similarly, PS at the BF was influenced by nearly all analyzed ANS metrics: time-domain (SDNN, *p* < 0.001; RMSSD, *p* < 0.001; meanNN, *p* < 0.001; pNN20, *p* < 0.001; pNN50, *p* = 0.004; xBRS, *p* = 0.001), frequency-domain (LFn, *p* < 0.001; LF/HF, *p* = 0.044), and selected nonlinear metrics (JSD_sym_, *p* = 0.015; MSEn, *p* = 0.003; ApEn, *p* < 0.001; SampEn, *p* = 0.048; FuzzyEn, *p* < 0.001). Changes in gain at the VLF were explained by EtCO_2_ (Supplementary Table S3), not by ANS metrics. Similarly, at BF, changes in gain were primarily explained by respiratory rate and EtCO_2_, with the impact of ANS metrics being negligible (Supplementary Table S3).

## Discussion

Consciously controlled breathing influences both the baroreflex and chemoreceptor activity, and alters cerebral autoregulation. Linear mixed-effects modeling showed that PS at VLF and BF, adjusted for EtCO_2_ and respiratory rate, was significantly affected by ANS activity during controlled breathing. These findings suggest that the withdrawal of ABP oscillations and their diminished control, reflected by reduced baroreflex sensitivity, lower JSD indices, and decreased HRV metrics, impacts PS. In contrast, linear mixed-effects modeling describing the relationship between gain and ANS metrics indicated that gain is primarily driven by respiratory parameters. This suggests that the direct effects of respiratory rate and EtCO_2_ on gain are stronger than those mediated through the ANS. Respiratory parameters should be considered when analyzing the relationship between ANS activity and cerebral autoregulation.

### Effects of controlled breathing on ANS activity

Slow, steady breathing increases venous return, enhances right heart filling, and elevates both stroke volume and cardiac output (Dick et al. 2014; Hsieh et al. 2003; Russo et al., 2017). Breathing modulates both parasympathetic and sympathetic neural drive to the heart. Altered breathing patterns, mediated by changes in CO_2_ levels through chemoreceptors in the brainstem, may interact with the autonomic cardiorespiratory control system (Lumb and Thomas 2020). Previous studies have shown that controlled breathing maneuvers induce substantial changes in ANS activity (Guzik et al. 2007; Paprika et al. 2014). In our study, we observed decreased ANS activity at higher respiratory rates, as reflected by reductions in xBRS and selected HRV metrics with LFn and LF/HF peaked at 6 bpm. A reduction in baroreflex function was associated with diminished cardiovascular oscillation at higher frequencies, as indicated by lower JSD indices, likely due to the shorter time available for expressing variability in this inertial system (Buchner et al. 2010). A random-effects meta-analysis has shown that paced breathing increases low-frequency HRV (Shao et al. 2024). The highest ANS activity observed at 6 bpm may be attributed to reduced chemoreflex overactivity during slower breathing (Spicuzza et al. 2000), which aligns with the reciprocal relationship between baroreceptors and chemoreceptors described in previous studies (Ponikowski et al. 1997).

### Nonlinear cardiovascular coupling and HRV metrics at higher respiratory rate

In our study, we investigated cardiovascular coupling during a controlled breathing maneuver using the JSD technique. Although cardiovascular oscillations have traditionally been analyzed using linear shift-invariant models, both cardiac and respiratory systems involve nonlinear dynamics (Baumert et al. 2015; Cairo et al. 2023). Tachograms are nonlinear and inhomogeneous time series (Sugihara et al. 1996). Consequently, nonlinear methods may complement traditional time- and frequency-domain approaches in better characterizing ANS activity and cardiovascular oscillations. One such approach is JSD, which involves coarse-graining observed time series into sequences of symbols (‘words’) to represent the system’s trajectory (Baumert et al. 2013). JSD has been successfully applied to analyze HR and SAP dynamics in various clinical contexts, including acute schizophrenia (Voss et al. 2010), dilated cardiomyopathy (Baumert et al. 2005), type 1 diabetes mellitus (Javorka et al., 2011b), and sleep apnea syndrome (Suhrbier et al. 2010). Changes in specific symbolic patterns have been associated with sympathetic activation and vagal withdrawal during the head-up tilt test (Porta et al. 2007a, b) and circadian rhythm (Porta et al. 2007a, b). In our study, a decrease in both JSD_diam_ and JSD_sym_ suggested that random changes dominated the diagonal and non-diagonal terms of the JSD matrix, indicating diminished cardiovascular coupling. Consequently, systemic control of ABP became less pronounced. The reduction in cardiovascular oscillations at higher frequencies likely reflects a shorter time window for expressing variability in this inertial system (Buchner et al. 2010). Furthermore, the observed reduction in baroreflex-like word types in the JSD matrix may also reflect decreased sympathetic activity with increasing respiratory rate. Kuo et al. demonstrated that a postural change from supine (associated with lower sympathetic activity) to standing (higher sympathetic activity) increases symmetric baroreflex-like patterns in healthy athletes, illustrating how JSD captures cardiovascular dynamics shifts (Kuo et al. 2003). The progressive changes in the symbolic word matrix observed in our study suggest that the system becomes more random and stochastic with a higher respiratory rate. Future studies should explore the potential of lagged joint symbolic analysis (Wessel et al. 2009) and joint conditional symbolic analysis (Porta et al. 2015) to monitor cardiorespiratory coupling across varying temporal schemes and latencies. Another nonlinear metric that is more resistant to noise than linear HRV indices is entropy (Solís-Montufar et al. 2020) (Bolea et al. 2014). The higher entropy observed at higher respiratory rates in our study reflects more complex or irregular heartbeat sequences (Li et al. 2015). It has also been hypothesized that complex cardiovascular oscillations are mainly sustained by a nonlinear vagal dynamic (Hager et al. 2025; Nardelli et al. 2020; Tulppo et al. 2001). Although not analyzed in this study, symbolic transfer entropy, which estimates transfer entropy through symbolization, could provide a robust alternative for quantifying information flow between time series (Staniek & Lehnertz 2008).

### Effects of controlled breathing on cerebral autoregulation

With increasing respiratory rate, we observed lower EtCO_2_ values. A reduction in PaCO_2_ is known to cause vasoconstriction and decrease CBv (Ito et al. 2003). This expected CBv response to CO_2_ was evident in our study, as CBv significantly decreased at 10 bpm and 15 bpm compared with spontaneous breathing. This cerebrovascular response to changes in PaCO_2_ is referred to as cerebral vasomotor reactivity (CVMR) (Czosnyka et al. 1993). CVMR functions as a counterregulatory mechanism to minimize changes in H^+^ concentration at the central chemoreceptor and, under normal physiological conditions, stabilizes the breathing pattern in response to perturbations in PaCO_2_ (Xie et al. 2005). Relative hypocapnia, secondary to likely V_T_ elevation when breathing at 10 and 15 bpm compared with 6 bpm and spontaneous breathing, was associated with improved cerebral autoregulation as reflected by higher PS at VLF (Aaslid et al. 1989; Birch et al. 1995). Conversely, PS at BF decreased with increasing respiratory rate. This reduction is likely not solely due to higher respiratory frequency but may be attributable to PS being estimated at a higher frequency. The decline in PS with increasing frequency is a well-established phenomenon, indicating that the efficiency of cerebral autoregulation diminishes at higher frequencies (Aaslid et al. 1989; Giller 1990; Zhang et al. 1998). Although the high-pass filter model of autoregulation predicts lower PS at higher respiratory frequencies, the influence of CO_2_ must also be considered. The effect of CO_2_ on PS, particularly at respiratory frequencies, remains incompletely understood (Lewis et al. 2012). While different approaches to quantifying cerebral autoregulation were not the focus of our study, we acknowledge that time-domain metrics are not limited to TFA analysis (Kostoglou et al. 2024b). Alternative time-domain methods include correlation-based indices (Czosnyka et al. 1996), autoregulation indices and the rate of regulation (RoR) (Aaslid et al. 1989), biophysical models (Ursino 1988), directional sensitivity (Aaslid et al. 2007), and multiple-input models (Panerai et al. 2000). In addition, nonlinear models have been used to characterize cerebral autoregulation, including Volterra-type models (Mitsis et al. 2002), artificial neural networks, and autoregressive support vector machines (Panerai 2004). Further research is needed to investigate the relationship between these methodologies and nonlinear ANS metrics.

### The impact of ANS activity on cerebral autoregulation

The cerebrovascular bed is richly innervated by adrenergic and cholinergic fibers of both extrinsic and intrinsic origins (Brassard et al. 2017). The effects of sympathetic and parasympathetic nerve activity on cerebral vessel diameter and CBF have been previously reviewed (Ainslie and Brassard 2014; Brassard et al. 2017; Koep et al. 2022; Mankoo et al. 2023). It has been hypothesized that the interaction between ANS activity and cerebral autoregulation reflects a dynamic regulatory mechanism that optimizes CBF control in response to pronounced arterial BP variability (Tzeng et al. 2010). From a dynamic systems theory perspective, it has been proposed that BRS and cerebral autoregulation cooperate to regulate CBF and achieve a stable “attractor state” (Witter et al. 2017). Experimental studies have demonstrated that ganglion blockade affects TFA metrics; specifically, autonomic blockade increases transfer function gain and decreases PS, suggesting impaired cerebral autoregulation (Zhang et al. 2002). Similarly, Ogoh et al. showed that cerebral autoregulation, assessed by the RoR, was attenuated after combined β-1 adrenergic and muscarinic cholinergic blockade (Ogoh et al. 2010). Tzeng et al. found an inverse relationship between the strength of cerebral autoregulation (quantified by RoR, autoregulatory index (ARI), and low-frequency gain) and both pharmacologically and spontaneously measured BRS (Tzeng et al. 2010). This was further confirmed by Witter et al., who observed that individuals with an attenuated BRS exhibited better cerebral autoregulation (Witter et al. 2017). Nasr et al. also reported an inverse correlation between cerebral autoregulation and BRS in healthy volunteers (Nasr et al. 2014). However, in contrast, a study on heart transplant recipients found that reductions in BRS were not associated with any changes in autoregulatory TFA metrics (Smirl et al. 2014). In our study, correlation between ANS metrics and cerebral autoregulation indices varied between spontaneous and controlled breathing, changing in both strength and direction with increasing respiratory rate, which made interpretation challenging. However, linear mixed-effects modeling revealed a significant influence of ANS activity on PS both at BF and VLF, whereas gain was primarily influenced by respiratory parameters. These findings highlight the importance of accounting for respiratory factors when analyzing the relationship between ANS activity and cerebral autoregulation. In complex physiological protocols, such as consciously controlled breathing, which affect both chemoreflex and baroreflex, advanced analytical approaches like linear mixed-effects models are advantageous.

### The utility of controlled breathing protocol in ANS and cerebral autoregulation studies

Our results demonstrated that a controlled breathing protocol is an effective, noninvasive tool for assessing complex autonomic reflexes and the modulation of cerebral autoregulation. Importantly, this protocol is easy to implement in both clinical and ambulatory settings. Controlled breathing is commonly used in research and clinical trials to reduce respiratory interference and standardize HRV measurement (DeBeck et al. 2010; Grossman et al. 1991; Piccirillo et al. 2004; Schaffer et al. 2014; Sin et al. 2010). However, HRV analysis can still be biased by the transition from spontaneous to consciously breathing (Sasaki & Maruyama 2014). In a study of 20 healthy volunteers, an increase in tidal volume (*V*_*T*_) was observed during controlled breathing at 15 bpm (mean *V*_*T*_: 594.7 ± 29.5 ml) compared to spontaneous breathing (mean *V*_*T*_: 534.6 ± 23.4 ml), despite similar respiratory rate (14.8 ± 0.7 bpm) during spontaneous breathing (Sasaki and Maruyama 2014). In our study, V_T_ was not measured. However, the observed decrease in EtCO_2_ at 15 bpm compared to spontaneous breathing, despite similar respiratory rate, suggests a likely increase in V_T_. Nevertheless, the influence of V_T_ on HRV analysis is still under investigation. Previous studies suggest that V_T_ has a limited effect on HRV, further supported by findings that its influence on fluctuations in R–R intervals is rather negligible (Brown et al. 1993; Cooke et al. 1998; Hirsch and Bishop 1981; Sasaki and Maruyama 2014). It has also been suggested that for short-term recordings, breathing protocols that incorporate stepwise changes in frequency, without stringent control of inspired volume, may provide the most efficient assessment of respiratory-mediated autonomic oscillations (Cooke et al. 1998). Further studies are needed to investigate the impact of V_T_ on ANS metrics. HRV analysis during a controlled breathing maneuver may also be influenced by changes in HR. In our study, both HR and ABP increased at higher respiratory rates. This response may reflect an increased work of breathing by participants, which could have implications for interpreting HRV data. Although changes in HRV are related to concurrent changes in HR (Monfredi et al. 2014), HRV should not be considered as a surrogate of HR. HRV analysis provides distinct and complementary information about cardiac autonomic regulation beyond what is offered by mean HR (Stauss 2014). Another aspect of controlled breathing is its ability to induce oscillations in ABP and CBv at respiratory frequencies, which are valuable for evaluating cerebrovascular dynamics using TFA. In the low-frequency range, achieving high coherence between ABP and CBv can be challenging. To address this, controlled breathing is often used to synchronize those oscillations (Gommer et al. 2010; Lang et al. 2001; Reinhard et al. 2003).

### Clinical utility of the relationship between ANS activity and cerebral autoregulation

Our findings have clinical relevance, as understanding the relationship between cerebral autoregulation and ANS activity may benefit patients with intracranial pathologies and cardiovascular diseases. Previous studies have shown that traumatic brain injury (TBI) can lead to dysautonomia, an acute autonomic overactivity syndrome (Baguley et al. 1999; Hasen et al. 2019). It has been suggested that impairment of cerebrovascular autoregulation and autonomic dysregulation are interdependent phenomena (Lavinio et al. 2008), potentially contributing to secondary insults ranging from reduced cerebral perfusion pressure to inflammatory responses (Sykora et al. 2009). Assessing the interaction between the ANS and cerebral autoregulation may also be important for patients with impaired vascular function, such as those with endothelial dysfunction, arteriosclerosis, or chronic hypertension. Studies have shown that spontaneously hypertensive patients, who are known to have reduced BRS, may exhibit better cerebral autoregulation than normotensive individuals (Hetzel et al. 2003). Tzeng et al. (2010) proposed that cerebral autoregulation and peripheral autonomic drive may reflect underlying neuroplasticity. This could explain the observed combination of reduced CBF and enhanced BRS in patients with chronic low blood pressure (Duschek et al. 2009).

### Limitations

Using transcranial Doppler CBv as a surrogate for CBF is considered valid, assuming that the diameter of the insonated artery remains constant during measurement (Kostoglou et al. 2024a, b; Panerai et al. 2023). A previous magnetic resonance imaging study indicated that, compared with the MCA diameter (2.9 ± 0.3 mm) at normocapnia (36 ± 3 mm Hg), hypocapnia (24 ± 2 mm Hg) did not result in significant diameter changes (2.9 ± 0.4 mm) (Serrador et al. 2000). A limitation of the current study is the absence of *V*_*T*_ monitoring, which may have influenced the observed cardiovascular responses. Although participants were instructed to avoid deep breathing, changes in *V*_*T*_ may have biased HRV analysis. In addition, participants were not instructed to refrain from intense physical activity prior to the study, which may affect cerebral autoregulation for up to 6 h (Burma et al. 2020; Kennedy et al. 2022). Although the study protocol included a brief recovery period between each of the respective respiratory rates, this washout period may have been insufficient to restore steady-state physiological conditions. Moreover, the controlled breathing sessions were not randomized, which may have introduced order effects into the dataset. Future studies may benefit from a protocol of paced breathing with dynamic end-tidal forcing and maintaining the end-tidal pressure of CO_2_ at a clamped level (Burma et al. 2021; Johnson et al. 2024). In our study, following the CARNET recommendation (Claassen et al. 2016a; Panerai et al. 2023), observations with coherence values below the critical values (adjusted for window size) were excluded from the TFA. However, this approach resulted in incomplete datasets for the respective respiratory rates. In this study, we used normalized values of HF and LF components of HRV, which reduced variability due to methodological differences. Further studies using alternative spectrum estimation methods is needed (Burr 2007). Finally, we did not analyze the influence of sex on the relationship between cerebral autoregulation and ANS activity, which limits the generalizability of our findings.

## Conclusions

The controlled breathing maneuver significantly impacted cerebral autoregulation and ANS activity. A higher respiratory rate led to diminished cardiovascular coupling, as revealed by JSD indices, and reduced systemic ABP control, as evidenced by lower xBRS and selected HRV metrics. The linear mixed-effects model, adjusted for EtCO_2_ and respiratory rate, showed that PS at VLF and BF were significantly affected by ANS activity, whereas gain was primarily driven by respiratory parameters.

## Supplementary Information

Below is the link to the electronic supplementary material.

Supplementary file1 (DOCX 437 kb)

## Data Availability

The data that support the findings of this study are available in the open-access repository Zenodo under the following link: 10.5281/zenodo.14005642.
