# Supplementary material for: Relationship between the autonomic nervous system and cerebral autoregulation during controlled breathing
Source: Eur J Appl Physiol. 2025 Aug 18;126(3):1357–74. doi: 10.1007/s00421-025-05933-9 (PMC13013261; doi:10.1007/s00421-025-05933-9)
Supplement: Supplementary file 1 — Supplementary file1 (DOCX 437 kb) [file 421_2025_5933_MOESM1_ESM.docx]

**Supplementary Data**

**Relationship between the autonomic nervous system and cerebral autoregulation during controlled breathing**

Agnieszka Uryga^1#^, Monika Najdek^1^, Piotr Urbański^2^, Magdalena Kasprowicz^1*^,
Teodor Buchner^3*^

1. Department of Biomedical Engineering, Faculty of Fundamental Problems of Technology, Wroclaw University of Science and Technology, Wroclaw, Poland
2. Clinical Department of Anesthesiology and Intensive Care, Faculty of Medicine, Wroclaw Medical University, Wroclaw, Poland
3. Faculty of Physics, Warsaw University of Technology, Warsaw, Poland

* Teodor Buchner and Magdalena Kasprowicz equally share senior authorship

#Corresponding author:

Agnieszka Uryga

Department of Biomedical Engineering, Wroclaw University of Science and Technology,

Wybrzeze Wyspianskiego 27,

50-370 Wroclaw, Poland

e-mail: agnieszka.uryga@pwr.edu.pl

tel. +48 71 320 46 65

ORCID ID: 0000-0001-8183-7643

**Joint Symbolic Dynamics (JSD)**

A bivariate vector *X* of length *n*, contains a time series of R–R intervals, and the SAP is defined as:

$X=\{\left[ x_{n}^{R-R},x_{n}^{SAP} \right]^{T}\}$ (S1)

where x $\mathbb{\in R}$. The formula presented by Baumert (Baumert et al., 2005) can be transformed into a bivariate symbol sequence named *S* as follows:

$S=\{\left[ s_{n}^{R-R},s_{n}^{SAP} \right]^{T}\}$ (S2)

where s $\in\{0,1\}$. Hence, $s_{n}^{R-R}$ is defined as 1 when $x_{n}^{R-R}-x_{n+1}^{R-R}<l^{R-R}$, and $s_{n}^{SAP}$ is defined as 1 when $x_{n}^{SAP}-x_{n+1}^{SAP}<l^{SAP}$. Otherwise, $s_{n}^{R-R}$ and $s_{n}^{SAP}$ are defined as 0. In the following, the threshold values are set to zero ($l^{R-R}$ = 0, $l^{SAP}$= 0). Considering word length three, this results in 64 different word types, which provide a statistically sufficient representation of the dynamics over a maximum of 30 minutes of beat-to-beat cardiovascular signals, covering the physiologically important time scale of respiratory dynamics (Baumert et al., 2015). The relative frequency of each of the 8×8 combinations of binary symbolic sequences $s_{n}^{R-R}$ and $s_{n}^{SAP}$ obtained from the bivariate time series *Z* can be written as a word distribution matrix *W,* from word type [000,000]^T^ to [111,111] ^T^:

$W= \left[ \begin{matrix} {R-R}_{000,} \\ \\ {R-R}_{111,} \end{matrix}\vdots\begin{matrix} {SAP}_{000,} \\ \\ {SAP}_{000,} \end{matrix}\begin{matrix} \cdots\\ \ddots\\ \cdots\end{matrix} \begin{matrix} {R-R}_{000,} \\ \\ {R-R}_{111,} \end{matrix}\vdots\begin{matrix} {SAP}_{111} \\ \\ {SAP}_{111,} \end{matrix} \right]$ (S3)

A schematic transformation of vector *X*, which contains a bivariate R–R interval and SAP samples, into matrix *W* is presented in Supplementary Figure 2. Word types were defined as 'dominant' if their probabilities of occurrence were greater than 0.05 and were defined as 'seldom' if they were less than 0.003. Based on the *W* matrix, it is possible to determine the relative frequency of baroreflex-like word types (JSD_sym_) and the relative frequency of patterns that are opposed to baroreflex behavior (JSD_diam_) (Baumert et al., 2015) as follows:

${JSD}_{sym}=\sum_{j=k=1}^{8} W_{j,k}$ (S4)

${JSD}_{diam}=\sum_{j=k=1}^{8} W_{j,9-k}$ (S5)

The sum of the relative frequency of baroreflex-like word types (JSD_sym_) and the sum of the relative frequency of patterns that are opposed to baroreflex behavior (JSD_diam_) have been established as high-sensitivity approaches for quantifying cardiovascular responsiveness to different stress manoeuvres (Baumert et al., 2013; Kabir et al., 2013).

**References**

Baumert, M., Baier, V., Truebner, S., Schirdewan, A., & Voss, A. (2005). Short- and long-term joint symbolic dynamics of heart rate and blood pressure in dilated cardiomyopathy. *IEEE Transactions on Bio-Medical Engineering*, *52*(12), 2112–2115. https://doi.org/10.1109/TBME.2005.857636

Baumert, M., Javorka, M., & Kabir, M. (2015). Joint symbolic dynamics for the assessment of cardiovascular and cardiorespiratory interactions. *Philosophical Transactions of the Royal Society A: Mathematical, Physical and Engineering Sciences*, *373*(2034). https://doi.org/10.1098/RSTA.2014.0097

Baumert, M., Javorka, M., & Kabir, M. M. (2013). Joint symbolic analyses of heart rate, blood pressure, and respiratory dynamics. *Journal of Electrocardiology*, *46*(6), 569–573. https://doi.org/10.1016/j.jelectrocard.2013.07.009

Kabir, M. M., Voss, A., Abbott, D., & Baumert, M. (2013). Joint symbolic dynamics as an effective approach to study the influence of respiratory phase on baroreflex function. *Annual International Conference of the IEEE Engineering in Medicine and Biology Society. IEEE Engineering in Medicine and Biology Society. Annual International Conference*, *2013*, 49–52. https://doi.org/10.1109/EMBC.2013.6609434


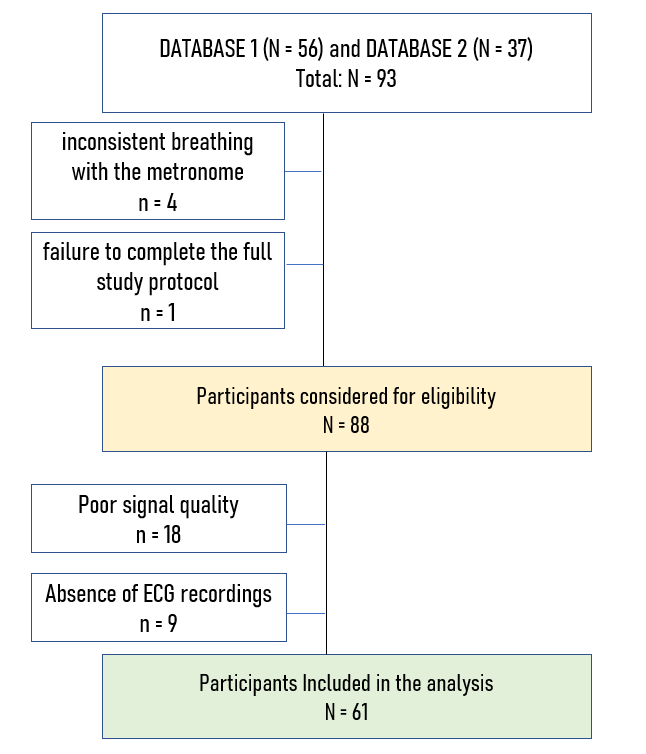


**Supplementary Figure 1**

The flow chart of the study.

**
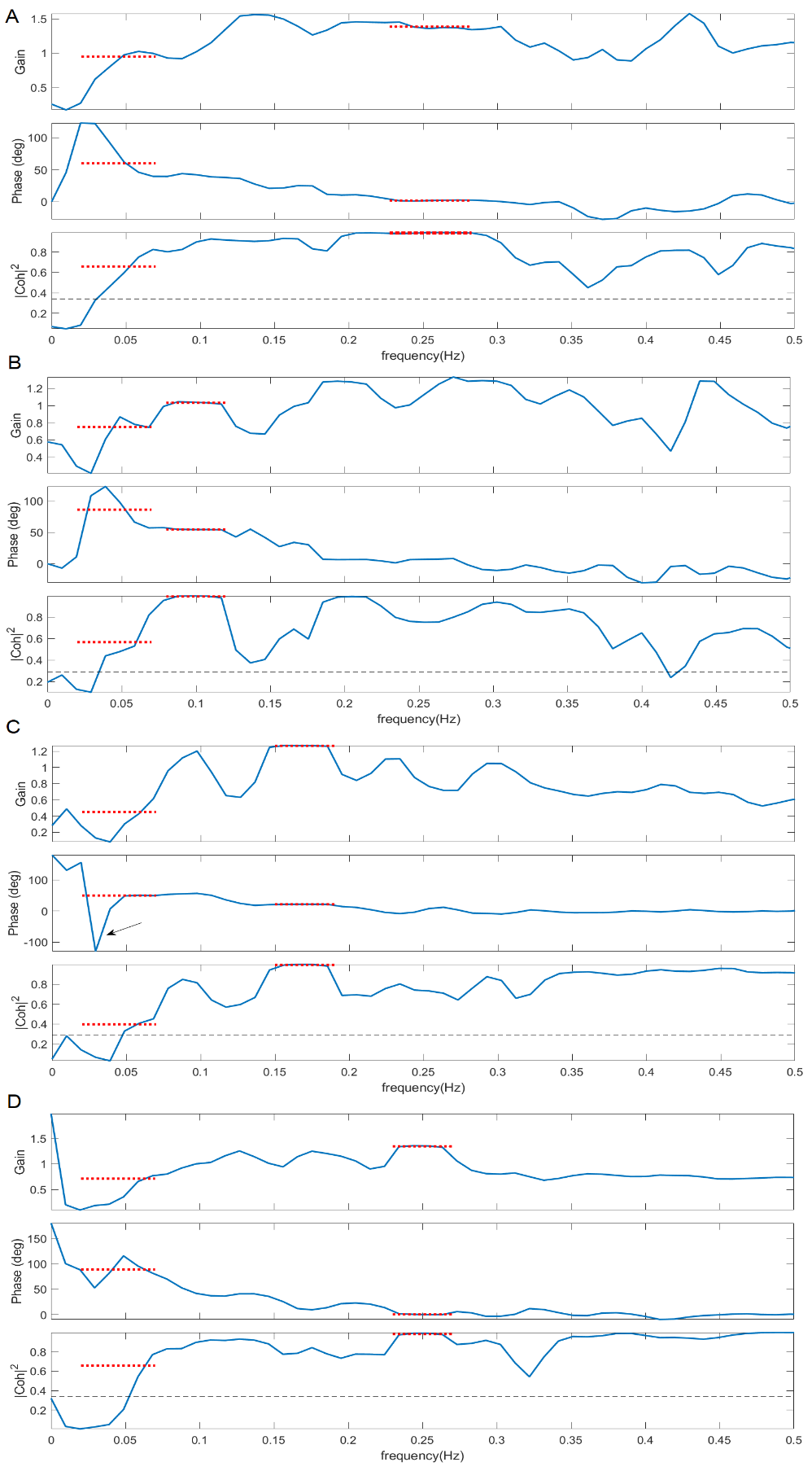
**

**Supplementary Figure 2**

An exemplary frequency-dependent curve of transfer function analysis (TFA) metrics: coherence, phase shift (PS), and gain is shown for spontaneous breathing (**A**) and controlled breathing at (**B**) 6 breaths/min **C**) 10 breaths/min **D**) 15 breaths/min. A coherence threshold (black dotted line) was applied using 95% confidence limits based on the number of windows. A negative PS for frequencies below 0.1 Hz was removed to avoid phase wraps (see arrow in panel C). TFA metrics were calculated in the 0.02–0.07 Hz frequency range (very low, VLF, first red dotted line in each panel) and at 0.1 Hz, 0.17 Hz, and 0.25 Hz ± 0.02 Hz (breathing frequency, BF, second red dotted line in each panel), which correspond to fixed breathing rates of 6, 10, 15 ± 1 breaths/min, respectively. For spontaneous breathing, the BF range was determined individually for each participant as the mean respiratory rate ± standard deviation.

**
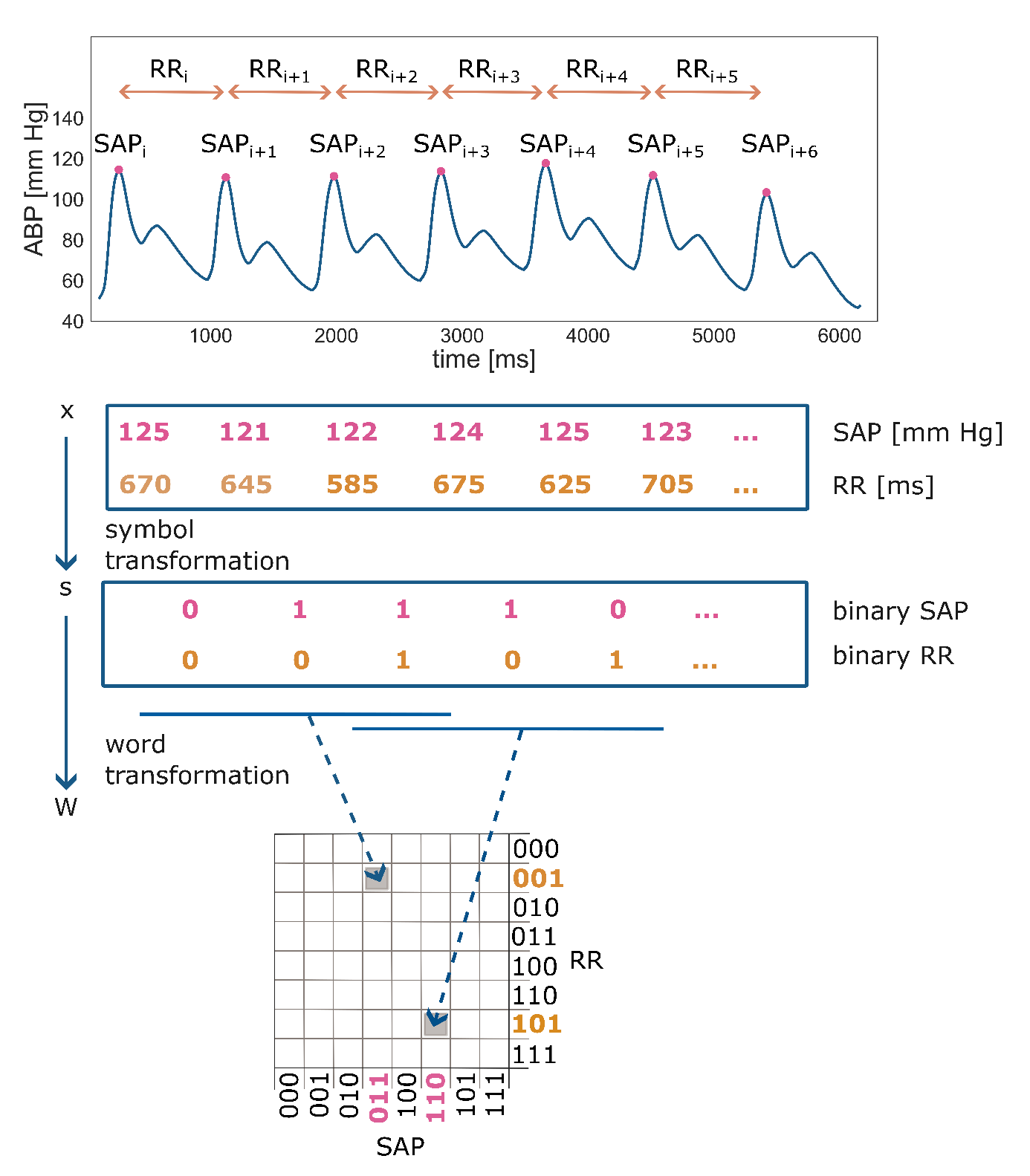
**

**Supplementary Figure 3**

Schematic representation of the joint symbolic analysis of R‒R intervals (R‒R; representing the duration of a ventricular cardiac cycle measured between two successive R waves) and a systolic arterial pressure (SAP) time series, using a binary symbolization procedure. Vectors *x* of beat-to-beat changes in R‒R and SAP were obtained from a noninvasive, continuous arterial blood pressure (ABP) signal, then transformed to vectors *s* composed of sequences of 1 (increases) and 0 (decreases or no changes), and finally into the word distribution density matrix *W*.

**Supplementary Table 1.** The linear mixed-effects model was used to assess the impact of the respiratory rate and end-tidal CO_2_ level (EtCO_2_) on the cerebral autoregulation metrics. Significant factors are marked in bold.

| **Parameter** | **respiratory rate**  **(bpm)** | | **EtCO_2_**  **(mm Hg)** | |
| --- | --- | --- | --- | --- |
|  | **F** | **p** | **F** | **p** |
| PS VLF [°] | 31.55 | **<0.001** | 30.61 | **<0.001** |
| Gain VLF [cm s^-1^ mmHg^-1^] | 3.03 | 0.085 | 87.45 | **<0.001** |
| Coherence VLF [a.u.] | 13.55 | **<0.001** | 220.20 | **<0.001** |
| PS BF [°] | 0.20 | 0.892 | 33.87 | **<0.001** |
| Gain BF [cm s^-1^ mmHg^-1^] | 87.07 | **<0.001** | 49.97 | **<0.001** |
| Coherence BF [a.u.] | 82.67 | **<0.001** | 592.41 | **<0.001** |

*Abbreviations:* PS, phase shift; VLF, very low frequency (0.02–0.07 Hz); BF, breathing frequency
(0.10; 0.17; 0.25 Hz ± 0.02 Hz)

**Supplementary Table 2.** The linear mixed-effects model for the total cohort of participants assesses the impact of end-tidal CO_2_ (EtCO_2_) and respiratory rate on variations in autonomic nervous system (ANS) metrics. Significant factors are highlighted in bold.

| **Parameter** | **respiratory rate**  **(bpm)** | | **EtCO_2_**  **(mm Hg)** | |
| --- | --- | --- | --- | --- |
|  | **F** | **p** | **F** | **p** |
| SDNN [ms] | 8.35 | **0.004** | 193.10 | **<0.001** |
| RMSSD [ms] | 0.51 | 0.478 | 111.78 | **<0.001** |
| MeanNN [ms] | 49.82 | **<0.001** | 702.88 | **<0.001** |
| pNN20 [%] | 0.21 | 0.645 | 297.63 | **<0.001** |
| pNN50 [%] | 1.26 | 0.263 | 92.42 | **<0.001** |
| xBRS [ms/mm Hg] | 0.06 | 0.815 | 59.55 | **<0.001** |
| LFn [a.u.] | 73.26 | **<0.001** | 400.61 | **<0.001** |
| HFn [a.u.] | 194.17 | **<0.001** | 34.56 | **<0.001** |
| LF/HF [a.u.] | 14.51 | **<0.001** | 1.07 | 0.303 |
| JSD_sym_ [a.u.] | 6.71 | **0.011** | 116.80 | **<0.001** |
| JSD_dia_ [a.u.] | 2.71 | 0.102 | 37.66 | **<0.001** |
| MSEn [a.u.] | 174.49 | **<0.001** | 240.60 | **<0.001** |
| ApEn [a.u.] | 371.83 | **<0.001** | 279.21 | **<0.001** |
| SampEn [a.u.] | 306.49 | **<0.001** | 57.08 | **<0.001** |
| FuzzyEn [a.u.] | 165.69 | **<0.001** | 222.42 | **<0.001** |

*Abbreviations*: SDNN, standard deviation of the R-R intervals; RMSSD, square root of the mean of the squared successive differences between adjacent R-R intervals; pNN20 and pNN50, proportion of R-R intervals greater than 20 ms or 50 ms, respectively; LFn, HFn, normalized power spectral density of the R-R interval time series in the low-frequency range (LF, 0.04–0.15 Hz) and the high-frequency range (HF, 0.15–0.40 Hz), obtained by dividing the respective power spectra by a total power (TP, 0.04–0.40 Hz); LF/HF; low-to-high frequency ratio; MSEn, multiscale entropy; ApEn, approximate entropy; SampEn, sample entropy, FuzzyEn, fuzzy entropy; xBRS, baroreflex sensitivity; JSD_sym_, the relative frequency of baroreflex-like word types; JSD_diam_, the relative frequency of patterns that are opposed to baroreflex behaviour

**Supplementary Table** **3.** The linear mixed-effects model for the total cohort of participants explains the variation in gain based on autonomic nervous system (ANS) metrics, adjusted for end-tidal CO_2_ (EtCO_2_) and respiratory rate. Significant factors are highlighted in bold.

|  | **ANS metric** | | | **respiratory rate**  **(bpm)** | | **EtCO_2_**  **(mm Hg)** | |
| --- | --- | --- | --- | --- | --- | --- | --- |
| **Cerebral**  **autoregulation** | **type of metric** | **F** | **p-value** | **F** | **p** | **F** | **p** |
| Gain VLF  [cm s^-1^ mmHg^-1^] | SDNN [ms] | 0.4 | 0.521 | 2.1 | 0.148 | 38.0 | **<0.001** |
|  | RMSSD [ms] | 1.4 | 0.238 | 2.7 | 0.107 | 60.3 | **<0.001** |
|  | MeanNN [ms] | 0.4 | 0.537 | 1.5 | 0.220 | 11.1 | **0.001** |
|  | pNN20 [%] | <0.1 | 0.839 | 3.0 | 0.087 | 31.7 | **<0.001** |
|  | pNN50 [%] | 0.7 | 0.409 | 2.7 | 0.102 | 66.4 | **<0.001** |
|  | xBRS [ms/mm Hg] | 0.8 | 0.377 | 2.9 | 0.094 | 64.6 | **<0.001** |
|  | LFn [a.u.] | 0.9 | 0.337 | 3.9 | 0.051 | 16.0 | **<0.001** |
|  | HFn [a.u.] | 1.8 | 0.177 | 0.3 | 0.581 | 88.2 | **<0.001** |
|  | LF/HF [a.u.] | 0.7 | 0.415 | 3.6 | 0.059 | 37.8 | **<0.001** |
|  | JSD_sym_ [a.u.] | 1.7 | 0.190 | 2.1 | 0.155 | 37.8 | **<0.001** |
|  | JSD_dia_ [a.u.] | 1.3 | 0.249 | 2.4 | 0.127 | 76.7 | **<0.001** |
|  | MSEn [a.u.] | 0.6 | 0.450 | 0.4 | 0.523 | 25.7 | **<0.001** |
|  | ApEn [a.u.] | >0.1 | 0.833 | 0.7 | 0.397 | 27.7 | **<0.001** |
|  | SampEn [a.u.] | 0.4 | 0.550 | 2.3 | 0.129 | 70.0 | **<0.001** |
|  | FuzzyEn [a.u.] | 0.2 | 0.647 | 0.9 | 0.357 | 35.6 | **<0.001** |
| Gain BF  [cm s^-1^ mmHg^-1^] | SDNN [ms] | >0.1 | 0.981 | 79.2 | **<0.001** | 18.7 | **<0.001** |
|  | RMSSD [ms] | 0.8 | 0.362 | 85.3 | **<0.001** | 35.0 | **<0.001** |
|  | MeanNN [ms] | 0.3 | 0.586 | 66.0 | **<0.001** | 6.2 | **0.014** |
|  | pNN20 [%] | 0.1 | 0.726 | 86.2 | **<0.001** | 17.6 | **<0.001** |
|  | pNN50 [%] | 1.3 | 0.252 | 85.2 | **<0.001** | 42.5 | **<0.001** |
|  | xBRS [ms/mm Hg] | 0.2 | 0.648 | 86.7 | **<0.001** | 30.2 | **<0.001** |
|  | LFn [a.u.] | 0.2 | 0.673 | 58.5 | **<0.001** | 18.5 | **<0.001** |
|  | HFn [a.u.] | 7.3 | **0.008** | 36.2 | **<0.001** | 53.8 | **<0.001** |
|  | LF/HF [a.u.] | 1.5 | 0.231 | 75.5 | **<0.001** | 19.6 | **<0.001** |
|  | JSD_sym_ [a.u.] | 2.6 | 0.108 | 81.7 | **<0.001** | 17.2 | **<0.001** |
|  | JSD_dia_ [a.u.] | 1.7 | 0.191 | 83.9 | **<0.001** | 47.8 | **<0.001** |
|  | MSEn [a.u.] | 4.2 | **0.042** | 28.7 | **<0.001** | 8.8 | **<0.001** |
|  | ApEn [a.u.] | 0.4 | 0.532 | 38.4 | **<0.001** | 22.2 | **<0.001** |
|  | SampEn [a.u.] | 2.2 | 0.142 | 51.1 | **<0.001** | 47.8 | **<0.001** |
|  | FuzzyEn [a.u.] | >0.1 | 0.861 | 43.3 | **<0.001** | 22.0 | **<0.001** |

*Abbreviations*: VLF, very low frequency [0.02–0.07 Hz]; BF, breathing frequency (determined individually for each participant during spontaneous breathing, and defined as [0.10; 0.17; 0.25 Hz±0.02 Hz] for controlled breathing); SDNN, standard deviation of the R–R intervals; RMSSD, square root of the mean of the squared successive differences between adjacent R–R intervals; pNN20 and pNN50, proportion of R–R intervals greater than 20 ms or 50 ms, respectively; LFn and HFn, normalized power spectral density of the R–R interval time series in the low-frequency range [LF, 0.04–0.15 Hz] and the high-frequency range [HF, 0.15–0.40 Hz], obtained by dividing the respective power spectra by the total power [TP, 0.04–0.40 Hz]; LF/HF; low-to-high frequency ratio; MSEn, multiscale entropy; ApEn, approximate entropy; SampEn, sample entropy, FuzzyEn, fuzzy entropy; xBRS, baroreflex sensitivity; JSD_sys_, relative frequency of baroreflex-like word types; JSD_diam_, relative frequency of patterns opposite to baroreflex-like word types
